# Development of a Single-Piece Sperm Counting Chamber (SSCC) for Aquatic Species

**DOI:** 10.3390/fishes7050231

**Published:** 2022-09-01

**Authors:** Jorge A. Belgodere, Yue Liu, Elizabeth L. Reich, Jason Eades, Terrence R. Tiersch, William Todd Monroe

**Affiliations:** 1 Department of Biological and Agricultural Engineering, Louisiana State University and Agricultural Center, Baton Rouge, LA 70803, USA; 2 Aquatic Germplasm and Genetic Resources Center, School of Renewable Natural Resources, Louisiana State University Agricultural Center, Baton Rouge, LA 70820, USA

**Keywords:** microfabrication, PDMS, zebrafish, goldfish, sperm, cell counting, open hardware

## Abstract

Accurate determination of sperm concentration in aquatic species is important for assisted reproduction and cryopreservation, yet is challenging as current counting methods are costly or not suitable for many species. The goal of this work was to develop a simple (single-piece and single-layer photolithography) sperm counting chamber (SSCC) for aquatic species. Goldfish (*Carassius auratus)* and zebrafish (*Danio rerio*) sperm were used for evaluation in the device, which was created with soft lithography. Four designs with different geometries were evaluated for counting accuracy. Open-corner and open-midpoint designs were the most accurate with no significant differences (*P* > 0.05) for most of the target sperm concentrations (0.5–1.0 × 10^8^ cells/mL). The open-corner design was not significantly different from the Makler^®^ counting chamber intended for human sperm cells (*P* = 0.6) but was significantly different from a hemocytometer (*P* < 0.001) intended for other cell sizes. Material cost of device production was USD 16 per unit, including photolithography supplies, glass slide and coverslip, and polydimethylsiloxane. The cost can be reduced to USD 2 per unit with repeated wafer casts. This device could be further refined for resin 3-D printing and sharing via open-hardware approaches and modified to best suit species specific applications.

## 1. Introduction

Evaluation and adjustment of sperm concentration are essential steps for reproducible utilization of assisted-reproduction technologies, such as artificial insemination and cryopreservation for development of germplasm repositories [1,2]. Sperm concentration can significantly affect cryopreservation at multiple steps in various ways [3]. For example, sperm concentration can affect post-thaw sample quality [4], including sperm motility, agglutination, and fertilization rates [5,6]. In addition, accurate evaluation and adjustment of sperm concentration can provide efficiency in use of valuable samples from imperiled or genetically characterized animals (e.g., transgenics, research mutants or genetically improved broodstock), especially with respect to small-sized species with limited sperm volumes. For example, 2 μL is a typical maximum volume of sperm sample that can be collected from single males of small-bodied live-bearing fishes [7] or zebrafish (*Danio rerio*). Overly concentrated samples tend to waste sperm when in vast excess relative to the number of eggs available for a single fertilization, whereas under-concentrated samples can compromise fertilization rates and waste eggs. Therefore, inefficient usage of limited samples due to unstandardized concentration evaluation can produce costly losses of valuable genetic resources (and related investment) and constitutes an uncontrolled variable in cryopreservation research and application [8,9].

Despite the importance of sperm concentration, most research laboratories and cryopreservation projects neglect standardized assessment because specialized hardware can be expensive, inaccurate without extensive training, and challenging to customize [10]. The most widely used devices for estimation of sperm concentration are hemocytometer chambers, due to their low cost (~USD 100) and high accessibility. However, hemocytometers were designed for counting of cells larger than fish sperm (e.g., 2–4 μm, [11] including common human cell lines that range from 30 to 100 μm in diameter [12]. As such, hemocytometers can be inaccurate when adopted for sperm counting without adequate training, because of problems such as cells stacking in multiple layers.

To address this, specialized counting devices such as Makler^®^ chambers have been designed to constrain chamber height (i.e., 10 μm) to ensure single layers of sperm cells are visualized, thus yielding more accurate results [13]. However, these specialized chambers are costly (average cost ~USD 800), and they must be replaced when the counting grids fade or become damaged. Automated instrumentation has been used to reduce human error, such as flow cytometry [14], microvolume spectrophotometry [15], and computer-assisted sperm analysis (CASA) [16]. However, these types of equipment were designed for sophisticated research and thus costs tens of thousands of dollars [17]. The use of automated equipment solely for evaluation of sperm concentration is cost prohibitive to most facilities that do not specialize in cryopreservation or reproductive biology [18]. In addition, these commercial products do not allow customization for the diverse sperm characteristics of aquatic species.

Previously, an inexpensive (material cost USD 0.07 per unit) and customizable device was developed as a Microfabricated Enumeration Grid Chamber (MEGC) to measure fish sperm concentration [17], and was fabricated using soft lithography techniques. Development of low-cost devices such as this could facilitate distribution and routine access to practical hardware to support much-needed standardization and reproducibility across the research community. For example, the UPSPERM used spectrophotometric measurements to determine both vertical velocity and sperm concentration in an open-source platform [19]. With decreasing cost and increasing resolution of consumer-grade 3-D printing in an open-hardware movement [20,21], this design can also be distributed as open hardware through file sharing and open fabrication [22,23]. However, several issues of the MEGC were identified by external testers, such as the requirement for complicated two-layer photolithography (involving alignment of photomask patterns) which prevented easy reproducible fabrication by users. In addition, the configuration of two separate pieces tended to introduce variations caused by improper handling by operators.

The goal of this work was to develop a simplified (single-piece and single-layer photolithography) sperm counting chamber (SSCC) to facilitate reproducible use and fabrication. Soft lithography was used to prototype the SSCC herein, but the design could be fabricated with resin 3-D printing as the resolution of these printers improves [23]. The PDMS device contained grid features for counting and wall features to contain the sample, which was pipetted directly onto the device followed by placement of a standard coverslip on top. The specific objectives were: (1) design and fabricate component prototypes; (2) evaluate operational utility and functionality, and (3) compare counting accuracy among performance prototypes and commercial products (i.e., Makler^®^ chamber and hemocytometer). The SSCC was developed and tested with sperm from aquatic species but can be used and customized for a broad range of animal species.

## 2. Materials and Methods

### 2.1. Design and Component Prototyping

**Figure 1.**
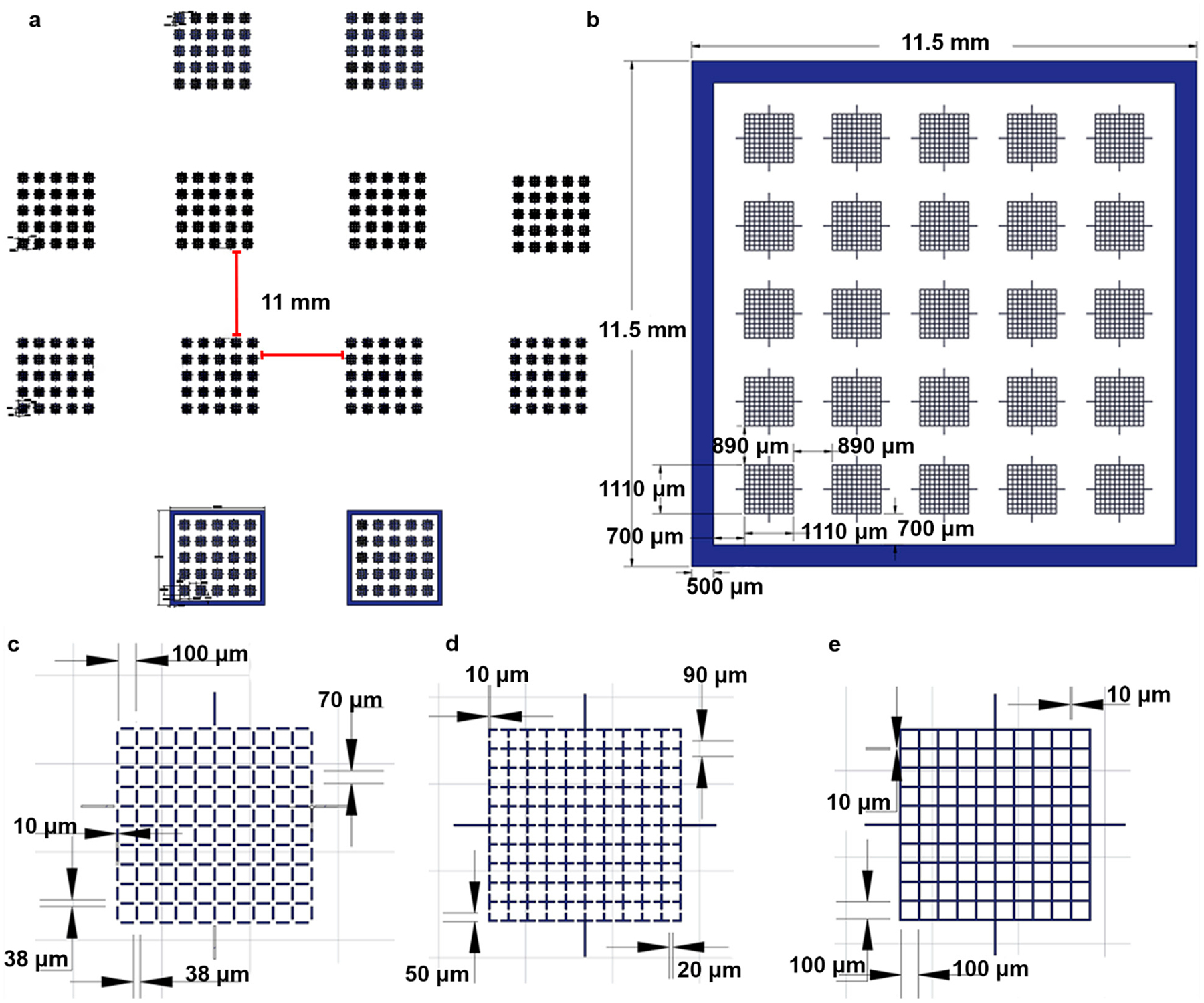
Renderings of the 2-dimensional design of SSCC device. Overall design used to create the photomask for fabrication of the SSCC chips (**a**). Each device contained 25 arrays in a 5 × 5 pattern (**a**,**b**), with the enclosed design incorporating a border surrounding the grids (**b**). Each grid contained 100 squares arranged in a 10 × 10 pattern (**c**–**e**) with each square measuring 100 μm (L) × 100 μm (W) × 10 μm (H). Three different grid designs were produced: open-corners (**c**), open- midpoint (**d**), and closed-grids (**e**). Only the closed-grid design surrounding the array (**b**).

The four different chips designs were sketched using computer-assisted design (CAD) software AutoCAD (Version Q.111, Autodesk, San Rafael, CA, USA) and oriented to create a photomask (Figure 1). Twelve assorted chips (4 × open-corner, 4 × open-midpoint, 2 × enclosed, and 2 × closed-grid lines) were able to fit in the photomask used to create the master mold (Figure 1a). Each chamber (Figure 1) contained 25 arrays, each of which contained 100 squares (100 μm × 100 μm) patterned by grid lines. The use of different closed and open configurations can affect uniformity of sperm distribution, and thus four different designs were prototyped for initial evaluation. It was hypothesized that including gaps in the grid lines would interconnect the squares and allow for sperm to distribute more evenly. Because zebrafish and goldfish sperm head sizes are <10 μm, a gap length of 20 μm facilitated sperm sample flow throughout the device and prevented blockage. The open-corner design reduced the grid line lengths from 100 μm to 70 μm and created a 20 μm gap in each corner. The open-midpoint had a 20 μm opening at the center of each grid line. Chamber perimeters were designed without (Figure 1a) and with (Figure 1b) enclosed walls (10 μm in height) for flow restriction. Additionally, three other grid patterns were designed, (Figure 1c–e), including an open-corner design (Figure 1c) with openings at square corners, an open-midpoint design (Figure 1d) with openings in the middle of the grid lines, and a closed-grid (Figure 1e) that fully enclosed squares. The enclosed and closed-grid line designs resembled the Makler^®^ and hemocytometer designs by using continuous grid lines. These two designs created isolated square cavities once the coverslip was placed on the surface. Each design was separated by ~10.5 mm to provide sufficient space when cutting chips from PDMS castings.

A previously described method by microfabrication with soft lithography was used for fabrication of component prototypes [24]. Geometrical patterns designed in CAD were used to create a photomask fabricated by a commercial provider (CAD/Art Services, Inc., Brandon, OR, USA). To fabricate the counting chamber, a master mold was patterned with photoresist SU-8 2025 (MicroChem Corporation, Newton, MA, USA) by use of a single-layer photolithography process. Briefly, SU-8 was spin-coated (Laurell Technologies Corporation, North Wales, PA, USA) on a silicon wafer (UniversityWafer, Inc., South Boston, MA, USA) with a thickness of 10 μm, UV cured (American Ultraviolet^®^, Lebanon, IN, USA) with the photomask, and developed (to remove unexposed SU-8) with SU-8 Developer (MicroChem Corp., Newton, MA, USA).

The wafer was cleaned with isopropyl alcohol (IPA, ≥99%, VWR International, Radnor, PA, USA) and deionized water (DI water, ≥17.8 mΩ), and dried with nitrogen gas. A 10:1 mixture (elastomer:curing agent) of Sylgard 184 polydimethylsiloxane (PDMS, DOW Corning, Inc., Midland, MI, USA) was cast onto the mold, degassed in a vacuum chamber, and cured in an oven at 65 °C for at least 2 h. The PDMS was demolded from the wafer, chambers were separated using a razor blade, and cleaned with IPA and DI water followed by drying with nitrogen.

For the bonding experiment, the non-feature sides of the PDMS chip were irreversibly bonded to glass slides using a plasma bonding technique. This creates an irreversible bond between PDMS and glass by excited surface molecules through exposure to plasma [25,26]. Once cured, the surface molecules return to their normal configuration and do not inhibit application of sperm samples. PDMS chips were treated with oxygen plasma using a Harrick Plasma Cleaner, PDC-32FG (Harrick Plasma, Ithaca, NY, USA) for 60 s at 1.8 W. Devices were rested overnight and cleaned with DI water prior to counting.

The PDMS surfaces were profiled using a Keyence VR-6100 (Keyence, Osaka, Osaka, Japan). To improve accuracy by enhancing reflectance, PDMS was dyed using black food coloring (Color Right Performance Food Coloring Set, Wilton, Darien, IL, USA). The four designs were profiled at 80-x magnification and processed using the VR-6000 series analyzer software (Ver. 4.2.2.54, Keyence, Osaka, Osaka, Japan). Average step height was calculated for four arrays on each device.

**Figure 2.**
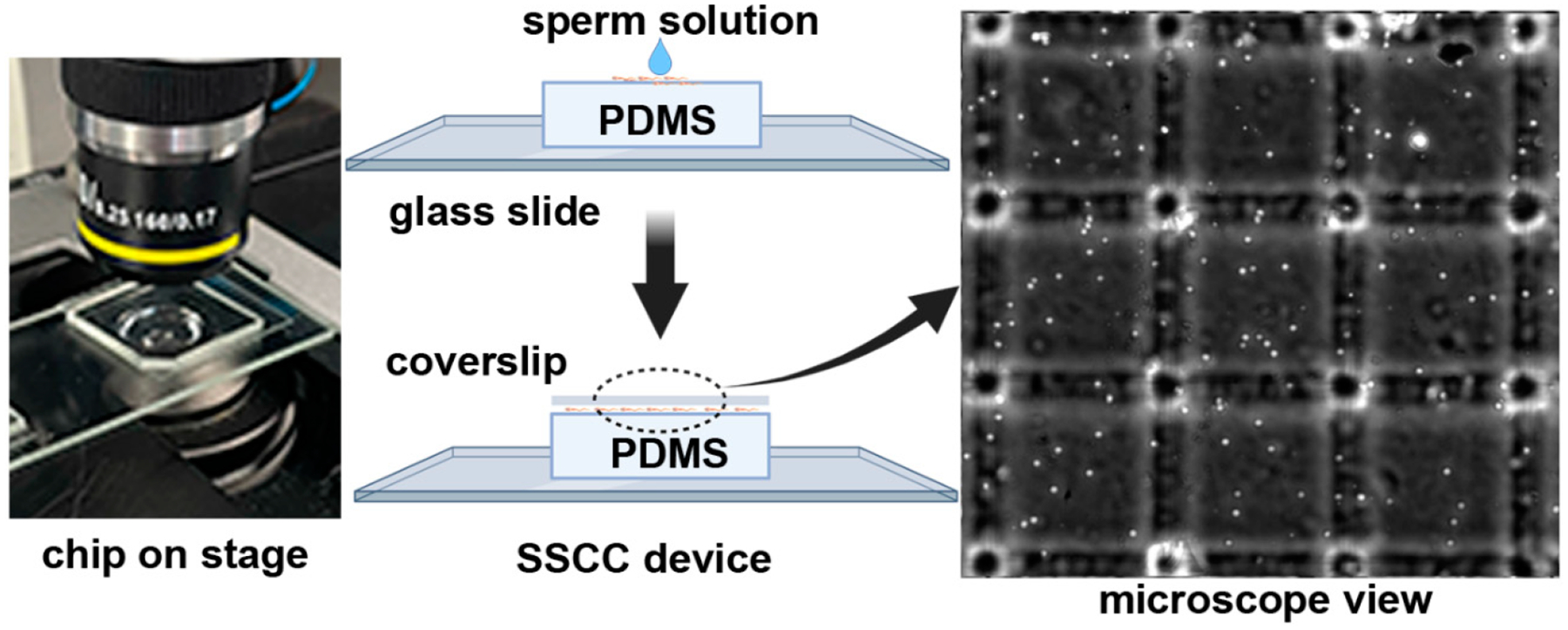
Operation of the SSCC device. The PDMS chip was placed on a microscope stage (**left**), sperm suspension was pipetted onto the surface, and a coverslip was applied to form a monolayer (**middle**). The 3 × 3 arrangement of chambers is shown at 100-× magnification (**right**). Created with BioRender.com.

The SSCC prototypes and coverslips (22 × 22 × 0.12–0.16 mm, AmScope, Irvine, CA, USA) were rinsed with DI, IPA, and dried with nitrogen or Kimwipes (Kimberly Clark, Irving, TX, USA). A 5 μL aliquot of sperm suspension was loaded to the center of the chamber by use of a micropipettor, followed by placement of a standard glass microscope coverslip on top (Figure 2). The chamber was placed on a microscope stage and viewed at 100-X magnification with a phase contrast filter (Nikon Eclipse Ti2, Melville, NY, USA). Sperm in five different arrays within each device were imaged and used for counting. The four corner and center squares in each array were counted and the average number of cells per square was calculated. The device surface was cleaned between samples by removing the coverslip and wiping the feature surface and coverslip with a Kimwipe. Once the surface was completely dry, the next sample was loaded and the coverslip replaced onto the device. The volume of a single square was 1 × 10^−7^ mL, calculated by multiplying the height (10 μm), width (100 μm), and length (100 μm). Sperm concentration (cells/mL) was estimated by dividing the average number of cells per square by 1 × 10^−7^ mL (volume per square).

### 2.2. Sperm Collection

Sperm samples from goldfish (*Carassius auratus*) and zebrafish were used to evaluate the functionality of prototypes. Protocols for the use of animals in this study were reviewed and approved by the Louisiana State University Institutional Animal Care and Use Committee. Adult zebrafish were maintained at the Aquatic Germplasm and Genetic Resources Center (AGGRC) within a recirculating system. Target values for water quality parameters were 20–26 °C, pH 8.5, and 14 h light:10 h dark photoperiod. Fish were fed to satiation twice daily with a dry food master mix (zebrafish.org/documents/protocols/pdf/Fish_Feeding). Additional water quality parameters that were monitored weekly and maintained within an acceptable range included: ammonia (0–1.0 mg/L), nitrites (0–0.8 mg/L) and nitrates (0–15 mg/L).

Male fish were anesthetized with 0.01% Tricaine methanesulfonate (MS-222, Western Chemical, Inc. Ferndale, WA, USA), placed with ventral side up on a moist sponge, and stripped by gently pressing the abdominal area with a finger. Sperm was collected into a 10-μL glass capillary tube (Drummond Scientific, Broomall, PA, USA), and immediately released into a 1.5-mL microcentrifuge tube containing Hanks’ balanced salt solution (HBSS, 0.137 M NaCl, 5.4 mM KCl, 1.3 mM CaCl_2_, 1.0 mM MgSO_4_, 0.25 mM Na_2_HPO_4_, 0.44 mM KH_2_PO_4_, 4.2 mM NaHCO_3_, and 5.55 mM glucose, pH 7.2) at 300 mOsm/kg. Sperm concentration was initially adjusted to 1.0 × 10^8^ cells/mL based on a Makler^®^ counting chamber (TS Scientific, Perkasie, PA, USA) as the base suspension, followed by dilution with ratios (base suspension:HBSS) at 3:1 (0.75 × 10^8^ cells/mL) and 1:1 (0.5 × 10^8^ cells/mL).

### 2.3. Evaluation of Operational Utility and Functionality

#### 2.3.1. Evaluation of Different Chamber Designs

Serial dilutions of goldfish sperm (1 × 10^8^, 0.75 × 10^8^, and 0.5 × 10^8^ cells/mL) were used to compare counting accuracy of four different chamber designs. Based on the results the prototypes with open-corner and open-midpoint grid lines were selected for additional evaluation of counting in different regions (four corner and center arrays within a chamber) within chambers to assess distribution homogeneity. Sperm in five different arrays were imaged and used for counting. The four corner and center squares in each array were counted and the average number of cells per square was calculated.

#### 2.3.2. Evaluation of the Feasibility of Repeated Use

Prototypes with open-corner and open-midpoint grid lines were used to determine the feasibility of reusing chambers for multiple counting. Zebrafish sperm at 1 × 10^8^ cells/mL was loaded to chambers, counted, the coverslip was removed from the device, a Kimwipe was used to wipe the surface clean of sample, and a new sample was applied to the device surface. This process was repeated for three times. Sperm in five different arrays were imaged and used for counting. The four corner and center squares in each array were counted and the average number of cells per square was calculated.

#### 2.3.3. Evaluation of the Feasibility of Bonding to Glass Slides for Durability

Plasma bonding of PDMS to glass slides is a common method used in fabrication of microfluidic devices to improve chamber durability and prevent deformation [27]. Zebrafish sperm at 1 × 10^8^ cells/mL was used to compare counting accuracy between bonded and unbonded open-corner and open-midpoints grid lines prototypes. Device usage and counting were performed as described above. Sperm in five different arrays were imaged and used for counting. The four corner and center squares in each array were counted and the average number of cells per square was calculated.

### 2.4. Accuracy Comparison of Performance Prototypes and Commercial Products

A standard zebrafish sperm solution was used to compare counting accuracy between SSCC prototypes and commercial products, i.e., Makler^®^ chamber and hemocytometer (WATSON Bio Lab, Japan). Each commercial device was operated following manufacturer recommendations. Zebrafish sperm was collected from 3–5 males as described above, and immediately placed into an Eppendorf tube with sperm extender, but no additional dilutions or adjustments were made. Samples were adjusted to known volumes following protocols established by the Zebrafish International Resource Center (ZIRC, zerbrafish.org) [28]. A 5-μL sample was loaded into the base piece of the Makler^®^ chamber, and a coverslip was placed on top. Sperm cells in ten squares within the first column were counted, and the averaged number of cells per square was multiplied by 1 × 10^5^, yielding sperm concentration per milliliter. No calibration was required, and additional counts were performed on other columns for a total of 5 counts.

Sperm samples were loaded into a hemocytometer and were counted following standard practices (hemocytometer.org). A 10-μL aliquot of sperm solution was loaded between the slide and hemocytometer, and capillary forces brought the sample into the viewing window. Cells were counted in the smaller squares (0.25 mm × 0.25 mm) of the top left, top right, bottom left, and bottom right of the larger enclosing square. Each smaller square contained 25 squares arranged in a 5 × 5 pattern. Any cells not completely within the counting regions were not counted. The average cells per small square, sum of all the cells counted divided by 4, was divided by the total volume in each small square. The volume of a small square was 1 mm (length) × 1 mm (width) × 0.1 mm (height) = 0.0001 mL. Hemocytometer counting was performed five times, with cleaning and reapplication of sample between each use. For the SSCC, sperm in five different arrays were imaged and used for counting. The four corner and center squares in each array were counted and the average number of cells per square was calculated.

### 2.5. Data Analysis

**Figure 3.**
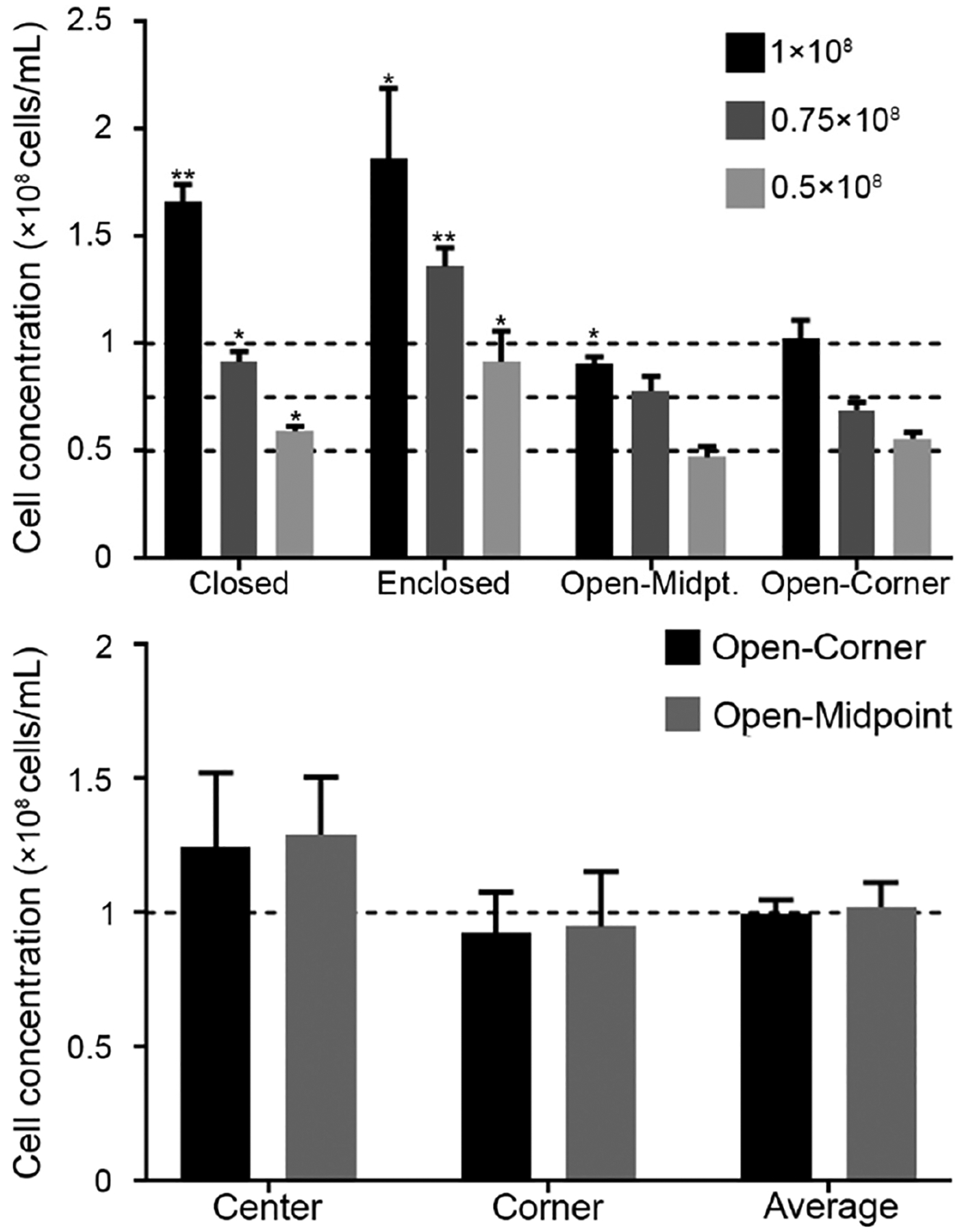
Comparison of four different designs for cell counts of serial dilutions of goldfish sperm at 1 × 10^8^, 0.75 × 10^8^, and 0.5 × 10^8^ cells/mL (*n* = 5; mean ± SEM) with one-sample *t*-test using the serial dilution concentrations as the hypothetical mean (**top**). Comparison of open-midpoint and open-corner designs for counting of zebrafish sperm (1 × 10^8^ cells/mL) (**bottom**) (*n* = 3) with two-way ANOVA for multiple comparisons (Dunnet), compared to Average * indicates *P* < 0.05 and ** indicate *P* < 0.01. The dashed line indicates solution concentrations.

**Figure 4.**
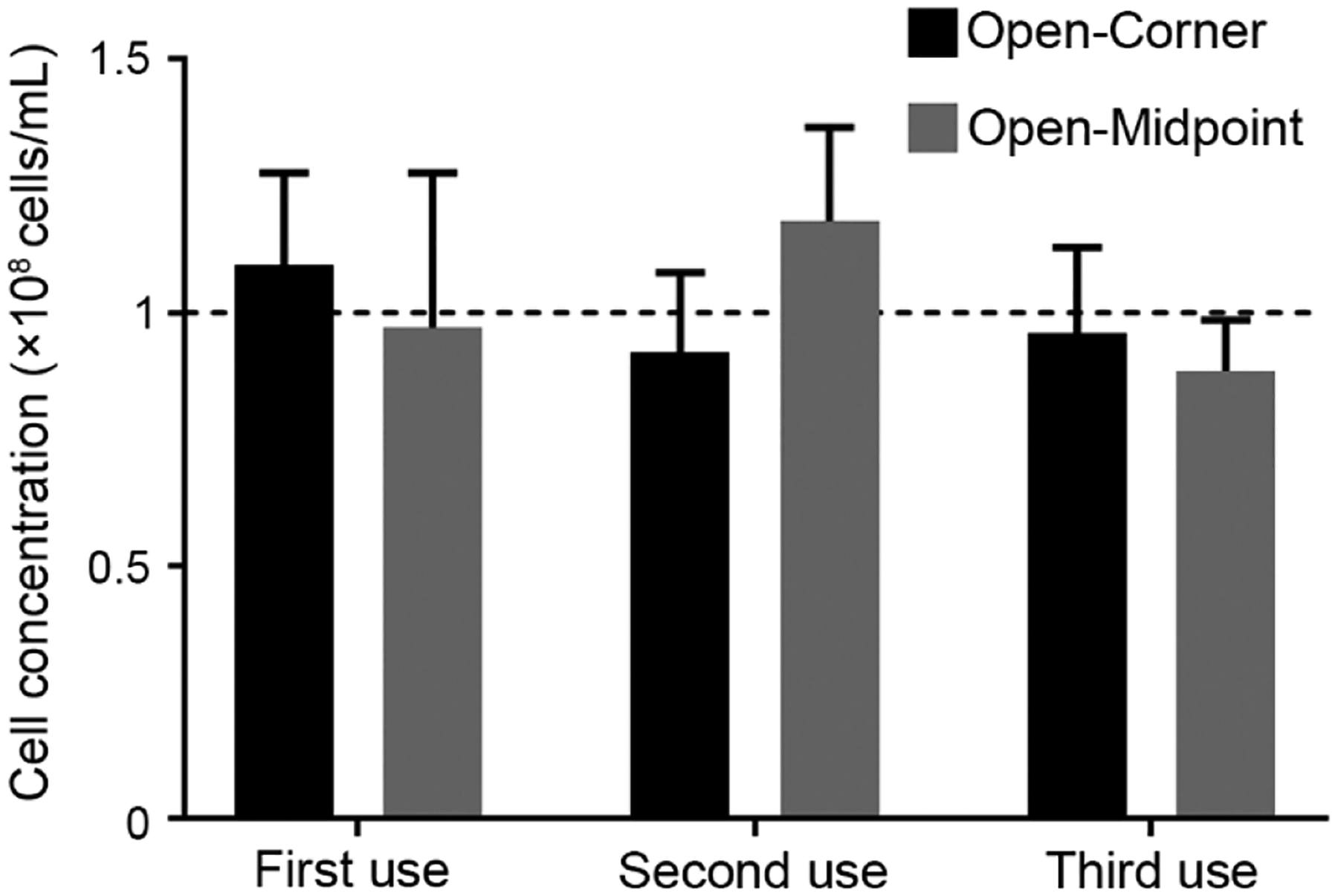
Repeatability of SSCC devices with open-corner and open-midpoint grid configurations for counting of zebrafish sperm (1 × 10^8^ cells/mL) (*n* = 5; mean ± SEM) with two-way ANOVA multiple comparisons (Dunnet), compared to first use. The dashed line indicates solution concentrations.

**Figure 5.**
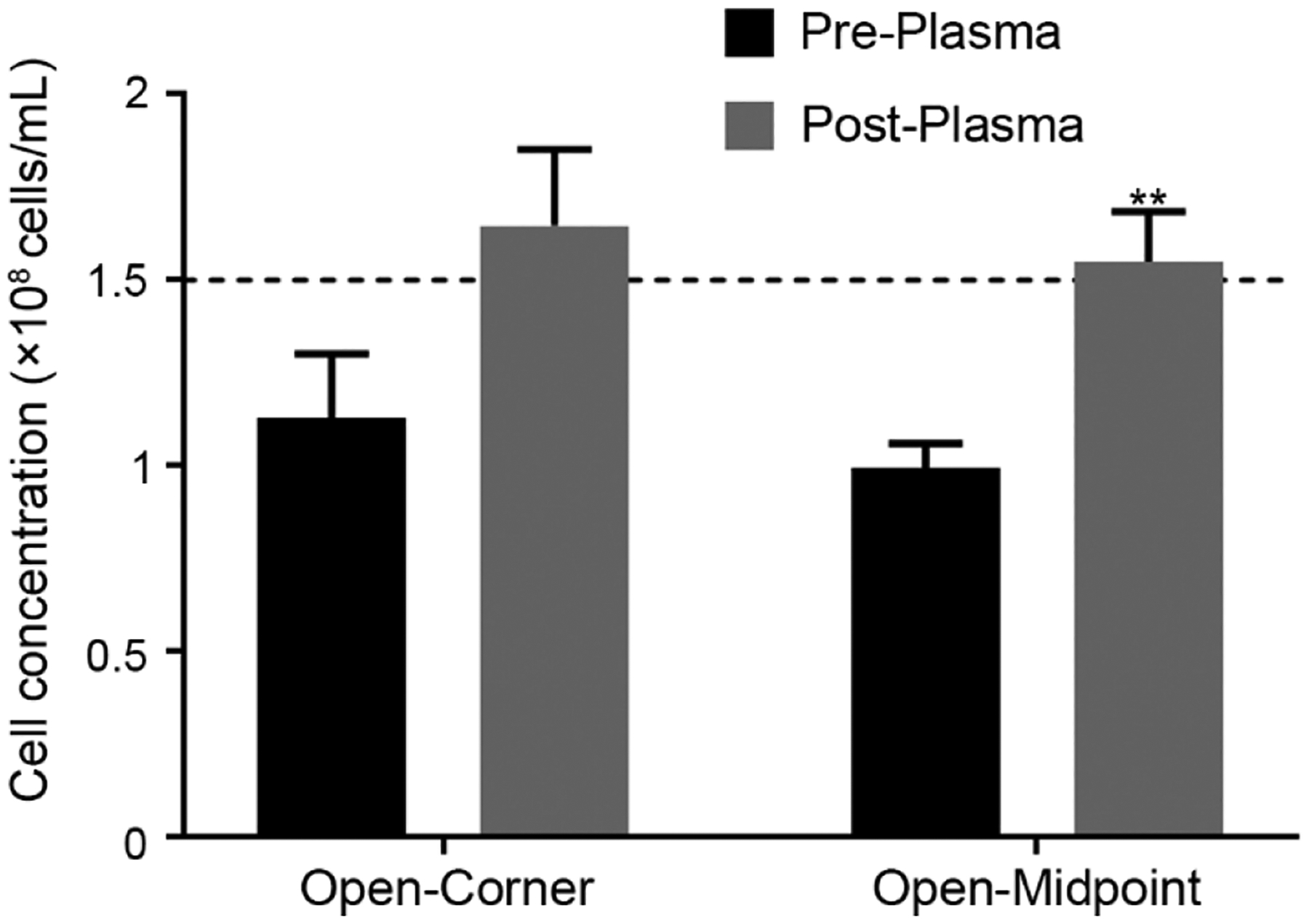
Comparison of the SSCC device with and without plasma bonding to a glass microscope slide for counting of zebrafish sperm (1.5 × 10^8^ cells/mL) (*n* = 5; mean ± SEM) with a *t*-test ** indicates *P* = 0.006. The dashed line indicates the solution concentration.

**Figure 6.**
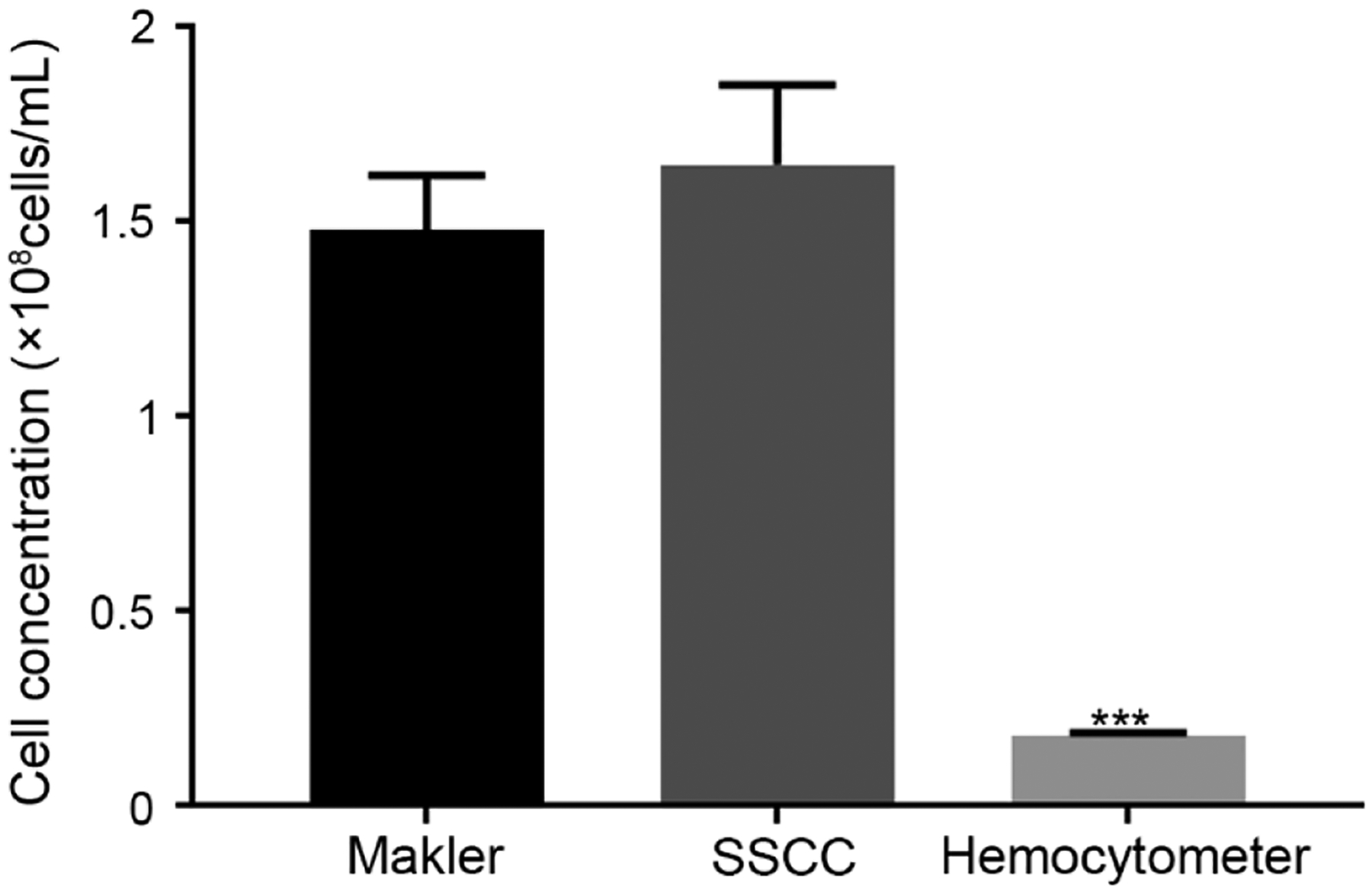
Performance comparison of a Makler^®^ counting chamber, SSCC device, and hemocytometer with zebrafish sperm (*n* = 5, Mean ± SEM) with two-way ANOVA multiple comparisons (Dunnet). *** indicates *P* < 0.001 when compared to the Makler^®^ and SSCC.

Statistical analyses were performed using GraphPad Prism (v8, GraphPad Software, San Diego, CA, USA). Two-way ANOVA multiple comparisons (Dunnet) (Figure 3 bottom, Figures 4 and 6), one-sample *t*-test (Figure 3 top) and standard *t*-test (Figure 5) were performed to determine significance of recorded values, indicated in each figure legend. *P*-values of < 0.05 were considered significant.

## 3. Results

### 3.1. Design and Component Prototyping

Photolithography produced a master mold with a designed height of 10 μm, preventing sperm stacking and facilitating monolayer formation. Initial prototypes consisted of a PDMS chip physically placed onto a microscope slide. PDMS castings were profiled to confirm resolution accuracy across different arrays. The open-corner design (6 ± 2 μm) had the lowest average grid line height, and the open-midpoint design (17 ± 1 μm) had the highest. The closed (14 ± 1 μm) and enclosed (12 ± 1 μm) designs were closer to the target grid line height of 10 μm. Feature resolution made it difficult to distinguish noise and artifacts of the PDMS surface from the grid lines. This likely led to deviations for average grid line height. Most importantly, no sperm compression or stacking was observed for any of the used devices and designs.

Early testing exposed issues with bubbles and debris between the glass slide and the non-feature side of the PDMS chip. Additionally, when handling the device, the PDMS was prone to delamination. Plasma bonding was an easy method that ensured the PDMS-glass interface was clean and no delamination occurred. The final working prototype was further evaluated using sperm collected from zebrafish and goldfish. Device assembly and usage is outlined in Figure 2.

### 3.2. Evaluation of Chamber Prototypes for Operational Utility and Functionality

#### 3.2.1. Comparison of Sperm Counting Accuracy for Each Design

The chambers with enclosed walls produced significantly higher concentrations for all three dilutions (*P* = 0.0255 for 1 × 10^8^, *P* = 0.0022 for 0.75 × 10^8^, and *P* = 0.0413 for 0.5 × 10^8^ cells/mL). Chambers with the closed-grid design produced significantly higher concentrations for all three dilutions (*P* = 0.0012 for 1 × 10^8^, *P* = 0.0232 for 0.75 × 10^8^, and *P* = 0.0174 for 0.5 × 10^8^ cells/mL) (Figure 3). The open-corner was the only design to not produce a significant difference between the measured and the target concentrations. While the open-midpoint design was superior to the enclosed chamber and closed grid designs, when used for the 1 × 10^8^ cells/mL solutions the counts were significantly lower (*P* = 0.0301). Based on these results, designs with open-corner and open-midpoint grid lines were chosen for subsequent experiments.

#### 3.2.2. Uniformity of Sperm Distribution within Chambers

Zebrafish sperm solutions were applied to the device surface and exhibited different flow patterns for sperm movement. After the coverslip was placed on the device, the sperm distribution varied based on the design. The closed-grid and enclosed designs prevented the flow of sperm and forced the cells to settle into the individual squares. Sperm that were not forced into squares would flow outwards towards the edge of the PDMS. For the enclosed design, sperm that reached the edge of the counting grid would “rebound” off the enclosed wall. Sperm rebounding occurred for approximately 1 min, or until the sperm finally settled into a square. This phenomenon likely led to lower accuracy and higher variability among replicates (Figure 3, top). The open-corner and open-midpoint designs had different flow profiles due to the openings in the grid lines. In each of these designs, sperm in solution would flow in one direction for ~10 s before settling. As such, only the open designs were evaluated further for sperm distribution. No significant difference was found between counts in the corner (*P* = 0.9) and center (*P* = 0.5) arrays compared to the average (Figure 3, bottom).

#### 3.2.3. Feasibility of SSCC Reusability

The SSCC devices were reused for triplicate counting and resulted in no significant (*P* = 0.7 for second use, and *P* = 0.8 for third use) differences between counts (Figure 4). Devices could be cleaned using either rinsing with DI water followed by drying with Kimwipes, or by only using a Kimwipe to wipe the surface clean. Cleaning the surface using a Kimwipe was much simpler and reliably removed all sperm from the surface. All data presented in Figure 4 used Kimwipes to clean the surface between counts. No changes in optical clarity were observed during the counting and no damage to grid lines or devices were noted.

#### 3.2.4. Determining the Effect of Plasma Bonding on SSCC Counting Accuracy

Preliminary prototypes demonstrated that plasma bonding would be advantageous by preventing handling issues, PDMS deformation, and enhancing optical clarity. When compared to non-bonded devices, bonded devices exhibited no changes in optical clarity and did not alter operation. Bonding prevented delamination of the PDMS from the glass and reduced contaminants at the glass-PDMS interface. Before plasma bonding, device assembly resulted in bubbles or particulates being seen at the interface. Accurately counting sperm in arrays amongst these visual artifacts was difficult. Cleaning between counts was also streamlined, as no delamination or shifting of the PDMS chip occurred during cleaning. No significant differences in counts made with unbonded and plasma-bonded devices were observed for the open-corner (*P* = 0.09) device (Figure 5). Counts from the open-midpoint device were significantly different between pre- and post-plasma bonding (*P* = 0.006). Due to performance differences between the two designs, the open-corner device was used for subsequent experiments.

### 3.3. Accuracy Comparison of Performance Prototypes and Commercial Products

The Makler^®^ counting chamber and the SSCC operated similarly, by pipetting the sample onto the device, followed by placement of a coverslip on top. The hemocytometer was operated by pipetting the sample into the chamber. The Makler^®^ and SSCC were each designed to produce a chamber height of 10 μm, and thus allowed for easy discrimination of individual sperm cells. The hemocytometer was designed for a height of 100 μm and resulted in sperm stacking within the counting window. Counting using the hemocytometer was difficult and resulted in significantly (*P* < 0.001) lower concentrations (estimated at 0.18 × 10^8^ cells/mL), when compared to the Makler^®^ counting chamber (1.48 × 10^8^ cells/mL) and the SSCC device (1.65 × 10^8^ cells/mL) (Figure 6).

## 4. Discussion

Practical evaluation and control of sperm concentration is critical for reliable operation germplasm of repositories. This becomes more important for imperiled species, and biomedical models from valuable research lines (e.g., zebrafish). Current standard devices, such as the Makler^®^ counting chamber, are costly and must be replaced or evaluated regularly to ensure continued accuracy. Hemocytometers have been used previously, but they lack accuracy when used for relatively small cells. Concerns over accuracy and use of the Makler^®^ counting chamber and hemocytometer have been reported in veterinary and research fields [29–32]. Thus, this work evaluated various chamber designs for the development of a reusable, low-cost counting device with similar or greater accuracy than currently used devices.

The SSCC designs were based on the general concepts of the Makler^®^ counting chamber and hemocytometer. Grid lines, designed with a height of 10 μm, prevented sperm stacking and allowed for equal distribution. Unlike the Makler^®^ chamber, the grid lines of the SSCC were physical barriers to sperm movement, more comparable to a hemocytometer. The layout of the master wafer included the four different designs with ample space between the grid patterns. Future wafers could be fabricated to include only a single design and reduce the spacing to increase the chip count per wafer. Photolithography is a common method used for microfluidic device fabrication and has shown no adverse effects when used with sperm of fish species [17,24,33,34].

Sputter coating of clear objects with a thin metallic layer is a common approach to increase surface reflectance of such substrates, when using laser-based profiling methods [35]. When SSCC chips were sputter coated, the surface features were lost, likely due to the strong vacuum used during the process. The thin grid line features (<10 μm in width and ~10 μm in height) appeared smooth or contained minimal feature resolution after sputtering. Optical profiling methods do not rely on reflectance of light from a small beam to create a 3-D surface scan. Rather, they illuminate the entire surface and collect surface data over a wide area based on the surface roughness [36]. To improve accuracy of optical profiling, black dye was incorporated into the PDMS prior to casting to provide sufficient contrast. The closed and open-midpoint designs were taller than the target height of 10 μm while the open-corner design was shorter. No correlation between device counting accuracy and wall height was observed (e.g., the open-corner design with the smallest chamber height and the open-midpoint design with the largest chamber height were equally accurate).

When evaluating the counting accuracy and precision of different SSCC designs, the tendency of sperm solution to flow across the grid was an important factor for consistent sample loading and dispersion. Features that restricted sperm flow (i.e., the closed-grid lines) resulted in an unequal distribution and elevated counts. The open-based designs held the sperm solution on the device through interfacial tension. These designs limited constriction of fluid flow but did not contain the applied solution. Sperm could move through any square across the array, allowing a uniform distribution. Furthermore, the distribution of sperm on the open-based designs allowed for precise counting without bias based on the region counted. Because the gaps between the chamber grid lines were large enough to allow for the sperm to move easily, there were fewer opportunities for accumulation across the array. Other reports have suggested Leucocytes [37] and mammalian cell types [38] distribution follow unique behaviors, e.g., non-Poisson, for, but none have tested zebrafish or goldfish sperm.

Current commercial counting devices are either disposable after a single use, which can be costly and wasteful, or are reusable, until feature loss prevents accurate counting. For the SSCC counting chamber to be considered low-cost, repeated use of a single fabricated device was important. The open-midpoint and open-corner designs showed no significant change in counts among three subsequent uses of the same sperm solution. Degradation of PDMS in microfluidics is not common [39] and the SSCC devices did not exhibit feature loss during collection of data.

A previous report indicated use of a two-piece PDMS chamber could lead to inaccurate counting due to deformation or “sagging” of the top chamber [17]. In the current design, this issue was circumvented in two ways: by having the PDMS component only on the bottom of the design, and by plasma bonding it to the glass slide base that constrained it from deforming. We found significant changes in counting accuracy when comparing before and after plasma bonding of the same device. Furthermore, plasma bonding is a common technique used in microfabrication and no reports of toxicity or danger to biological species have been reported [40]. Plasma bonding the non-feature side to glass prevented deformation, preserving counting accuracy. Additionally, these devices were easier to handle, clean, and transport after bonding to a microscope slide. This method was useful for data collection and for use on traditional microscopes.

The SSCC prototype produced counting results similar to the Makler^®^ chamber, but different from those of the hemocytometer. While the Makler^®^ chamber and hemocytometer are each commercially made products with fixed heights, the SSCC can be adaptable based on the target species requirements. By increasing the photoresist height in the spin coating step, the square wall height can be adapted to meet the wide range of sperm sizes across different species. Thus, the ease of customization for the height in the SSCC is a major advantage compared to comparable alternatives when considering the diversity of sperm sizes [41,42].

The production costs to develop and fabricate the SSCC device involved two separate steps: development of the wafer, and casting of the PDMS chips. Subsequent PDMS castings (at least five) were able to be made without noticeable loss to the resolution and features. There are few published reports to our knowledge on the number of PDMS casts that would produce feature loss, but the application of a silane layer has been suggested to improve PDMS demolding and retention of master wafer features [43]. The materials used to create the SSCC (PDMS and glass slides) were optically transparent, and non-toxic to biological species [40]. It cost approximately USD 16 per unit when only performing a single PDMS cast onto the master wafer (Table 1). If additional casts are performed (e.g., ten) the cost per unit is reduced to USD 2. Compared to the Makler^®^ chamber (~USD 800) and hemocytometers (~USD 100), the SSCC is more affordable with no sacrifice in counting accuracy. The costs are further reduced when additional PDMS casts are performed and when considering that microscope slides and coverslips are reusable. Additional modifications can lead to smart-phone integration for inexpensive and mobile sperm analysis without the need for bulky lab equipment [44].

## 5. Conclusions

The importance of accurate sperm counting is a critical and underappreciated aspect of aquatic species reproductive biology. Developing a simple and cost-effective counting device is paramount. Using commercial devices as starting points, the SSCC device was designed to address these concerns. Four grid alternatives were prototyped using photolithography to create PDMS chips bonded to glass microscope slides. This created robust features with high resolution that withstood processing and assembly. The open-corner and open- midpoint designs were the most accurate, distributing sperm cells evenly across the surface, and maintaining accuracy during repeated usages after plasma bonding to glass. The SSCC prototype operated similarly to the commercial devices and was as accurate or better when used to count zebrafish sperm.

For the present study, soft lithography techniques were used for prototyping purposes. Specialized equipment was required to produce the master wafer molds. However, the evaluated designs can be used in open-hardware applications [45–47]. Advancements in 3-D printing have allowed for fabrication of microfluidic devices with consumer-level machines that cost <USD 400 [22,23]. It is possible to adapt the designs developed here and fabricate 3-D printed SSCC devices that would have similar material costs, but much lower equipment costs. Finally, the chamber height and other features can be modified for other species and applications.

## Figures and Tables

**Table 1.** Per-unit cost analysis for the SSCC device. Supplies were calculated based on material needed to produce one master wafer and casting of a single SSCC device or a batch of 12 devices.

Item **	Cost (USD) *	Unit
Glass slide	8	72 pack
Glass coverslip	5	100 pack
PDMS	126	500 g + 50 g
Photomask	177	1 mask
Wafer	475	50 pack
SU-8	497	500 mL
SU-8 developer	133	4 L
Material costs for 1 cast	16	per unit
Material costs for 10 casts	2	per unit

* Prices were sourced from vendors in June 2022.

** 4 mL of SU-8 were required per wafer, 25 mL of SU-8 developer were required per wafer, and PDMS casting on wafers was 2 mm thick.

## Data Availability

All data is contained within the article.
